# RBBP5 drives cancer stem cell-like characteristics by suppressing the hippo signaling pathway through WNT5A downregulation in breast cancer

**DOI:** 10.1080/15384047.2026.2756150

**Published:** 2026-11-07

**Authors:** So-Jeong Moon, Young-Hyeon Kye, Gu Kong

**Affiliations:** a Department of HY-KIST Bio-convergence, College of Medicine, Hanyang University, Seoul, Republic of Korea; b Department of Pathology, College of Medicine, Hanyang University, Seoul, Republic of Korea

**Keywords:** RBBP5, cancer stem cell, breast cancer, tumor initiation, Hippo pathway

## Abstract

**Background:**

Cancer stem cells (CSCs) are a major contributor to breast cancer recurrence and therapeutic resistance. This study investigates the oncogenic role of Retinoblastoma Binding Protein 5 (RBBP5) in driving CSC-like properties in breast cancer.

**Methods:**

Genetic alterations in RBBP5 and their correlation with patient survival were analyzed using multiple breast cancer cohorts, including METABRIC, TCGA, and Kaplan-Meier (KM) plotter datasets. The impact of RBBP5 on CSC-like traits was assessed through tumorsphere formation and ALDEFLUOR assays, and in vivo xenograft models. To elucidate the underlying molecular mechanism, RNA sequencing (RNA-seq) was performed.

**Results:**

Elevated RBBP5 expression was significantly associated with poor prognosis in breast cancer. (METABRIC: OS, *p* = 0.007; DFS, *p* = 0.049; TCGA: OS, *p* < 0.001; KM plotter: RFS, *p* < 0.001). RBBP5 overexpression enhanced tumorsphere-forming capacity, CSC-like population and tumor-initiating cell frequency. RNA-seq results revealed an enrichment of gene sets associated with negative regulation of cell growth, invasiveness, and stem cell population in RBBP5-knockdown cells. RBBP5 knockdown upregulated WNT5A, a non-canonical Wnt ligand, leading to Hippo pathway activation and subsequent suppression of YAP signaling. Pharmacological inhibition of YAP1 abrogated RBBP5-induced CSC-like properties, while suppression of MST1/2 in RBBP5-knockdown cells restored YAP activity and CSC traits.

**Conclusions:**

These findings indicate that RBBP5 promotes CSC-like properties in breast cancer by repressing WNT5A-mediated Hippo signaling. We suggest RBBP5 as a potential biomarker for poor prognosis and propose that targeting YAP or activating the Hippo pathway could represent a promising therapeutic approach for patients with RBBP5-overexpressing tumors.

## Introduction

Breast cancer is the second leading cause of cancer-related deaths among women in the United States.^1^ Although mortality rates have been declining due to advances in treatment, many patients still suffer from recurrence.^2^ One of the main mechanisms for tumor recurrence is the presence of cancer stem cells (CSCs), which possess several characteristics including self-renewal capacity, differentiation potential, and tumorigenic ability.^3^ Therefore, therapeutic strategies targeting CSCs and their associated signaling pathways have been actively investigated to prevent tumor recurrence and overcome drug resistance in cancer patients.^4^

Retinoblastoma-binding protein 5 (RBBP5), a component of the Mixed Lineage Leukemia complex, has been shown to play oncogenic roles in various cancer types. RBBP5 is associated with poor prognosis and promotes cell proliferation in diverse cancer types including hepatocellular carcinoma.^5–8^ Its association with epithelial-mesenchymal transition (EMT) has also been reported in breast, prostate, and colorectal cancers.^9–11^ However, little is known about the role of RBBP5 in tumor initiation and CSC-like properties, particularly in breast cancer.

Numerous studies have elucidated pathways involved in CSC maintenance and function. The Wnt/β-catenin, Notch, Hedgehog, JAK-STAT, and Hippo pathways are well-established contributors to CSC properties in various types of cancer, including breast cancer.^3^ ^,^ ^12^ Among these pathways, the Hippo pathway plays diverse roles in various biological processes, including cell proliferation and differentiation, tissue regeneration, and embryogenesis. Dysregulation of the Hippo pathway is frequently observed and contributes to tumor initiation, progression, metastasis, therapy resistance, and poor prognosis in various cancer types, including breast, lung, and pancreatic cancers.^12–14^ Several studies also have demonstrated that the dysregulated Hippo pathway contributes to stem cell-like properties of various types of cancer, such as breast, ovarian, and esophageal cancers.^15–17^

In this study, we explore the oncogenic role of RBBP5 in breast cancer, focusing on its impact on CSC activity and the underlying molecular mechanisms. Our findings demonstrate that RBBP5 promotes CSC-like characteristics through regulation of WNT5A expression and subsequent modulation of the Hippo signaling pathway. This research not only enhances our understanding of RBBP5’s function in breast cancer but also evaluates its potential as a prognostic biomarker for breast cancer patients.

## Results

### RBBP5 overexpression correlates with unfavorable outcomes in breast cancer patients

In our previous study, we incidentally observed that among the components of the WRAD complex, RBBP5 exhibited more frequent dysregulation compared to WDR5 and DPY30, and showed a similar level of dysregulation to ASH2L in a significant proportion of breast cancer patients (Figure 1a).^18^ This unexpected finding prompted further investigation in the current study. Specifically, RBBP5 amplification was detected in approximately 26% of cases from the METABRIC dataset and 13% from the TCGA dataset. Notably, RBBP5 amplification positively correlated with its mRNA expression (*r* = 0.48, *p* < 0.001, in METABRIC; *r* = 0.49, *p* < 0.001, in TCGA; Figure 1b).

**Figure 1.**
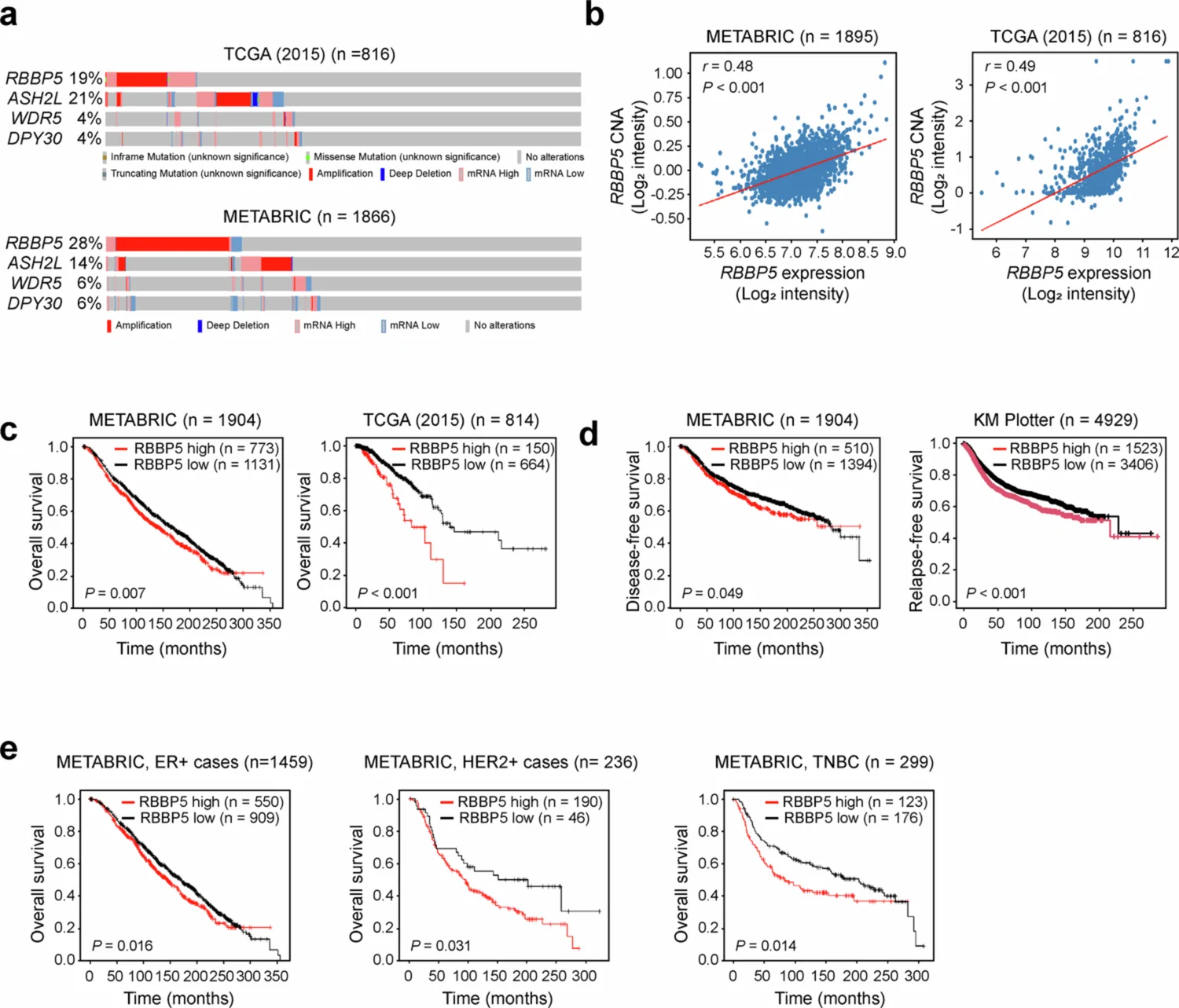
RBBP5 overexpression Correlate with Unfavorable Outcomes in Breast Cancer Patients. a Oncoprints illustrating the genetic alterations of WRAD complex members across the indicated breast cancer datasets. b Scatter plot showing the correlation between RBBP5 copy number alterations (CNA) and gene expression levels in the indicated breast cancer datasets. Pearson’s correlation coefficient (*r*) was calculated, and *p*-values were determined using one-way ANOVA. c and d Kaplan–Meier survival analysis of breast cancer patients stratified by RBBP5 expression levels in the indicated datasets. (c) Overall survival. (d) Disease-free and relapse-free survival. *p*-values were calculated using the log-rank test. e Overall survival analysis of patients with the indicated breast cancer subtypes in the METABRIC dataset. *p*-values were determined by the Kaplan–Meier method and the log-rank test.

To investigate the impact of RBBP5 expression on breast cancer patients' survival, we conducted survival analyzes using publicly available datasets, including METABRIC,^19^ TCGA,^20^ and Kaplan-Meier (KM) plotter.^21^ High RBBP5 expression was associated with poor overall survival in both METABRIC and TCGA datasets (*p* = 0.007, in METABRIC; *p* < 0.001, in TCGA; Figure 1c). Additionally, patients with high RBBP5 expression exhibited poorer disease-free survival in METABRIC and KM plotter datasets (*p =* 0.049, in METABRIC; *p* < 0.001; in KM plotter, Figure 1d).

Further analysis of overall survival based on breast cancer subtypes including estrogen receptor-positive (ER+), human epidermal growth factor receptor 2-positive (HER2+), and Triple-negative breast cancer (TNBC) in the METABRIC dataset revealed poorer overall survival in patients with high RBBP5 expression across all three subtypes (*p =* 0.016, ER+; *p* = 0.031, HER2+; *p* = 0.014, TNBC; Figure 1e). Collectively, these results demonstrate that increased RBBP5 expression is associated with poor prognosis in breast cancer patients.

### RBBP5 drives the acquisition of cancer stem cell phenotypes in breast cancer

Given that cancer stem cells (CSCs) are a major contributor to poor prognosis and recurrence in cancer patients, we investigated the impact of RBBP5 on the acquisition of CSC-like properties in breast cancer cells using tumorsphere formation and ALDEFLUOR assays.

According to the METABRIC data, we did not observe differential expression of RBBP5 among the three subtypes (ER+, HER2+, TNBC). Therefore, we did not restrict our selection of breast cancer cell lines to a specific subtype for this study (Fig. S1a). Based on information from CCLE data and our screening data (Fig. S1b and c), we selected tamoxifen-resistant MCF7 (TamR), ZR-75-1, and HCC-1143 cells to generate shRNA-mediated RBBP5 knockdown cells. For RBBP5 overexpression experiments, we utilized MCF7, T47D, and HCC-1428 cells (Fig. S1d).

Overexpression of RBBP5 in MCF7, T47D, and HCC-1428 cells increased the population with high enzymatic activity of ALDH1, an indicative marker of breast cancer stem cells,^22^ while RBBP5 knockdown in TamR, ZR-75-1, and HCC-1143 cells resulted in a reduction of this population (Figure 2a). Furthermore, tumorsphere-forming ability was enhanced in RBBP5-expressing cells, while RBBP5-knockdown reduced the capacity to form tumorspheres (Figure 2b).

**Figure 2.**
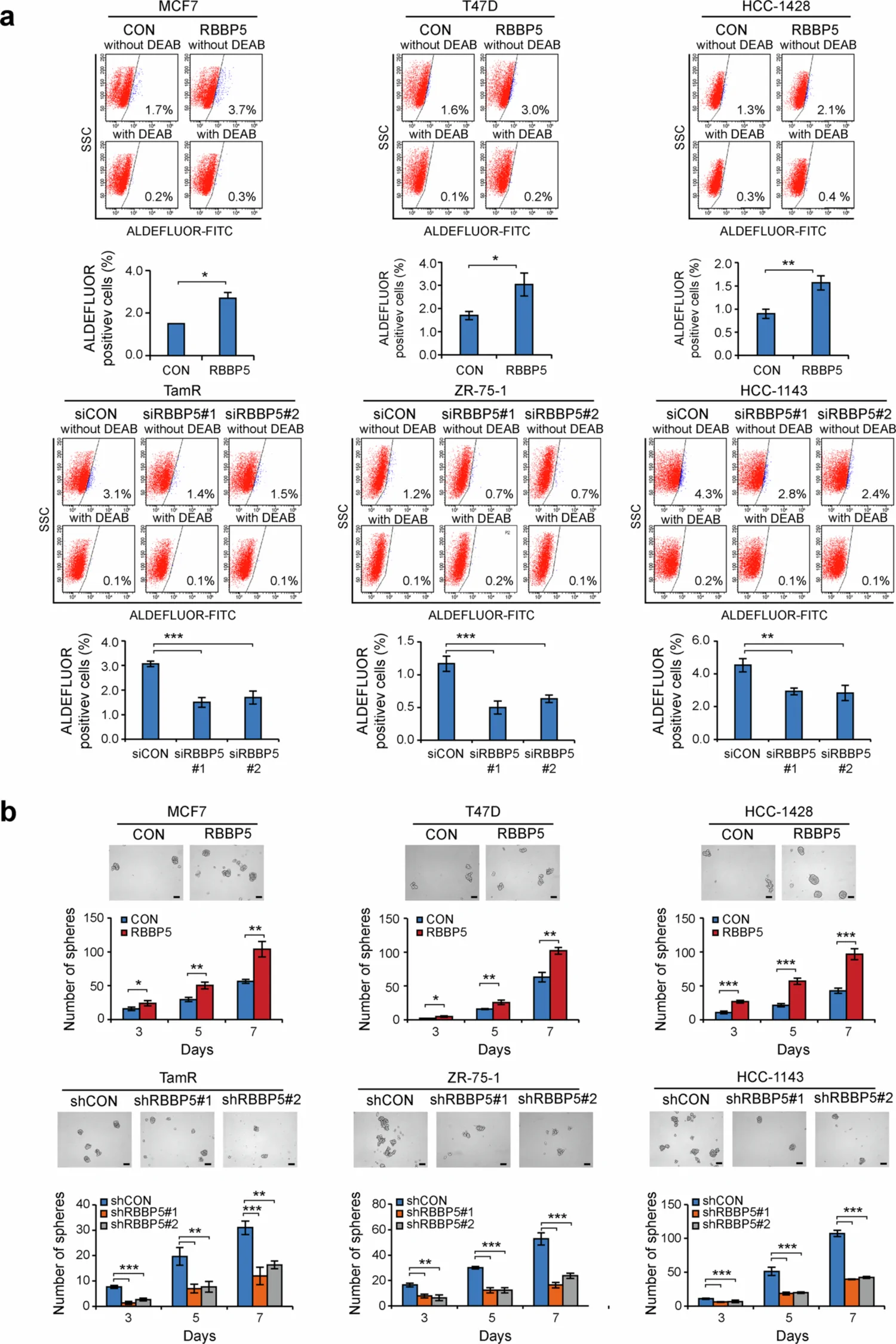
RBBP5 Drives the Acquisition of Cancer Stem Cell Phenotypes in Breast Cancer. a Representative flow cytometry plots and quantification of cell populations with high ALDH activity in the indicated cells. Cells were incubated with the ALDEFLUOR reagent in the presence or absence of the specific ALDH inhibitor diethylaminobenzaldehyde (DEAB). The gate for ALDEDLUOR-positive cells was set based on the DEAB-treated control. Data are presented as means ± SD from three independent experiments. *p*-values were determined using two-tailed Student’s *t*-tests for MCF7, HCC-1428, and T47D cells or one-way ANOVA followed by a post hoc LSD test for tamoxifen-resistant MCF7 (TamR), HCC-1143, and ZR-75-1 cells. *p < 0.05, **p < 0.01, ***p < 0.001 versus controls (CONN or siCON). CONN, control; RBBP5, RBBP5 overexpression; siCON, siRNA control; siRBBP5, RBBP5 siRNA; with DEAB, DEAB-treated samples; without DEAB, samples not treated with DEAB. b Tumorsphere formation assays were conducted to assess the self-renewal capacity of CSCs in the specified cell lines. Data represent the mean ± SD from three independent biological replicates. *p*-values were evaluated using two-tailed Student’s t-tests (for MCF7, HCC-1428, and T47D) or one-way ANOVA followed by an LSD post hoc test (for TamR, HCC-1143, and ZR-75-1). *p < 0.05, **p < 0.01, ***p < 0.001 compared to the control groups (CONN or shCON). CONN, empty vector; RBBP5, RBBP5 overexpression; shCON, shRNA control; shRBBP5, RBBP5 knockdown. Scale bar 100μm.

These in vitro findings were corroborated by in vivo xenograft models using NOD/SCID mice. Increased tumor-initiating cell (TIC) frequency was observed in mice bearing RBBP5-overexpressing MCF7 cells compared to control MCF7 cells, while the opposite results were observed in RBBP5-knockdown ZR-75-1 cells (Table 1). Additionally, a higher tumor growth rate was observed in RBBP5-overexpressing MCF7 cells compared to control cells, whereas RBBP5 knockdown reduced tumor growth rate in ZR-75-1 cells (MCF7: *p* = 0.033; ZR-75-1: *p* = 0.001; RM ANOVA; Fig. S2a, S2b and S2c). Collectively, these results demonstrate that RBBP5 influences CSC-like characteristics in breast cancer cells.

**Table 1.** RBBP5 enhances in vivo tumor-initiating ability.

Cell type	Cell number for injection	Days		
		28 day	35 day	42 day
MCF7 CONN	2500	0/7	0/7	0/7
	5,000	0/7	0/7	2/7
	10,000	0/7	2/7	5/7
TIC frequency			1/56,102 (1/14,230-1/221,181)	1/12,704 (1/6,094-1/26,484)
MCF7 RBBP5	2,500	0/7	0/7	0/7
	5,000	0/7	3/7	5/7
	10,000	1/7	4/7	7/7
TIC frequency		1/117,429 (1/16,707-1/825,374)	1/13,145 (1/6,250-1/27,645)	1/4,584 (1/2,560–1/8,207)
*p-*value			0.07	0.033
		Days		
Cell type	Cell number for injection	24 day	26 day	28 day
ZR-75-1 shCON	5,000	0/7	0/7	3/7
	10,000	0/7	3/7	5/7
	50,000	4/7	7/7	7/7
TIC frequency		1/86,351 (1/32,996-1/225,982)	1/19,645 (1/9,891-1/39,018)	1/8,250 (1/4,133-1/16,465)
ZR-75-1 shRBBP5	5,000	0/7	0/7	0/7
	10,000	0/7	0/7	2/7
	50,000	0/7	3/7	4/7
TIC frequency		1/86,351 (1/32,996-1/225,982)	1/125,004 (1/40,987-1/381,247)	1/54,956 (1/24,137-1/125,127)
*p-*value			0.0057	0.0006

Note: Control (CONN) and RBBP5-overexpressing (RBBP5) MCF7 cells (2,500–10,000 cells) and control (shCON) or RBBP5-knockdown (shRBBP5) ZR-75-1 cells (5,000–50,000 cells) were orthotopically injected into the mammary fat pads of NOD/SCID mice. Tumor-initiating cell (TIC) frequencies were estimated using L-Calc software (STEMCELL Technologies, http://www.stemcell.com).

### RBBP5 facilitates YAP1 activation by suppressing WNT5A expression

To elucidate the underlying mechanism of altered CSC activity in breast cancer cells with differential RBBP5 expression, we conducted RNA sequencing analysis using control and RBBP5-knockdown HCC-1143 cells. Gene set enrichment analysis (GSEA) of RNA-seq results revealed that gene sets associated with oncogenic properties, such as mammary stem cells and angiogenesis, were enriched in control HCC-1143 cells. Conversely, gene sets related to negative regulation of cell growth, invasiveness, and stem cell population were enriched in RBBP5-knockdown HCC-1143 cells (Figure 3a and b). Several differentially expressed genes (DEGs) involved in cancer initiation and progression were validated through quantitative real-time polymerase chain reaction (qRT-PCR) (Figure 3c and d). Among the DEGs, we observed that WNT5A expression was elevated in response to RBBP5 knockdown. Since RBBP5 is well characterized as a core component of the MLL complex that promotes H3K4me3 deposition at promoter regions of certain genes to drive transcriptional activation,^23^ ^,^ ^24^ we reasoned that the regulation of WNT5A by RBBP5 might be independent of H3K4me3. To address this possibility, we examined RBBP5 occupancy and H3K4me3 levels at the WNT5A promoter by Chromatin Immunoprecipitation quantitative real-time PCR (ChIP-qPCR) assay. No statistically significant differences in RBBP5 occupancy or H3K4me3 levels were observed between control and RBBP5-overexpressing HCC1428 cells, despite slight increases in both RBBP5 occupancy and H3K4me3 enrichment in RBBP5-overexpressing cells. Conversely, RBBP5 knockdown in HCC1143 cells significantly decreased RBBP5 occupancy at the WNT5A promoter by approximately 50% compared with the control (*p* < 0.001), whereas H3K4me3 enrichment showed a modest downward trend that did not reach statistical significance (Fig. S3a and S3b). These findings indicate that RBBP5-mediated repression of WNT5A may not primarily depend on direct modulation of promoter-associated H3K4me3, suggesting an alternative H3K4me3-independent mechanism.

**Figure 3.**
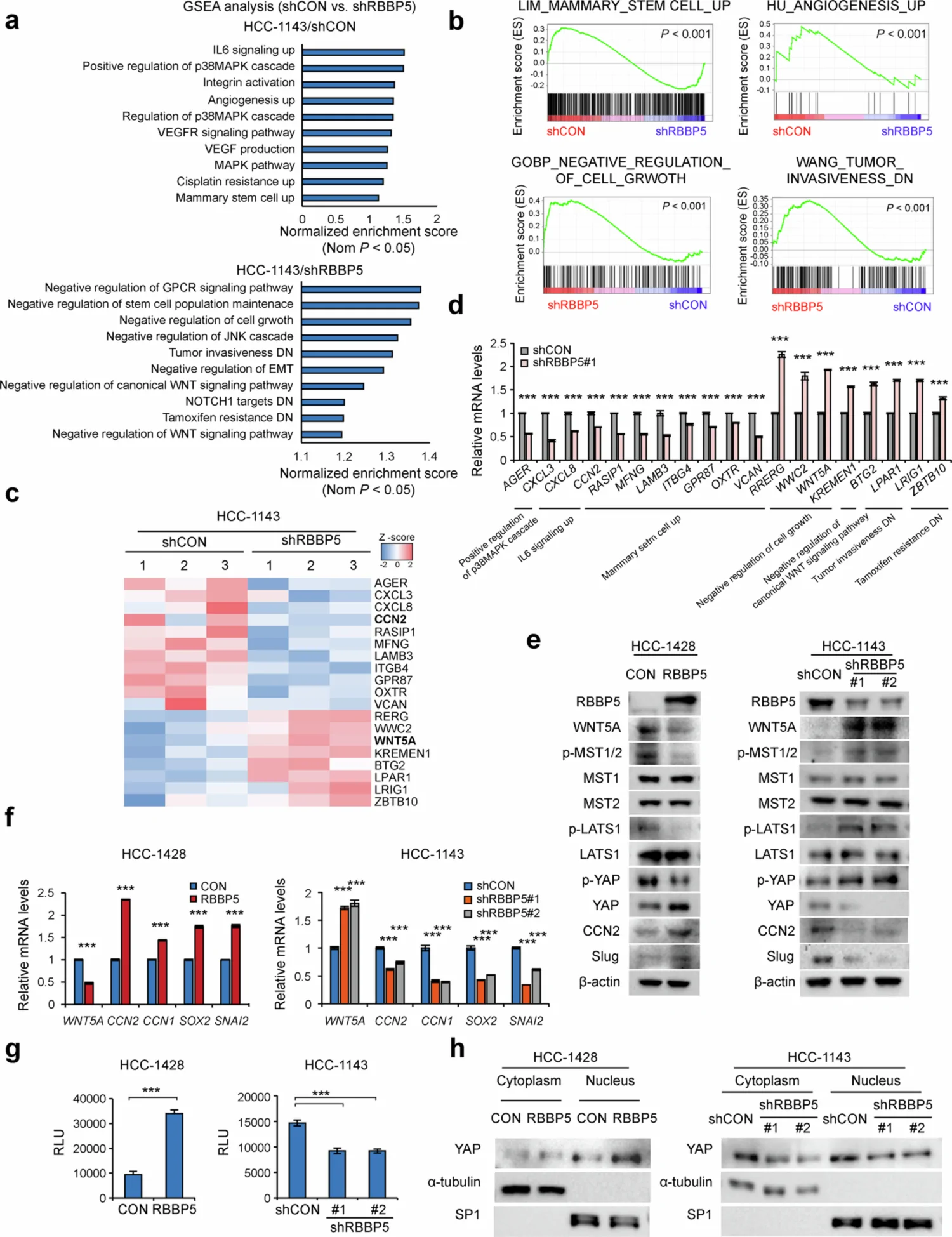
RBBP5 facilitates YAP1 activation by suppressing WNT5A expression a and b Gene set enrichment analysis (GSEA) of RNA-seq data revealing signaling pathways and gene signatures altered by RBBP5 knockdown in HCC-1143 cells. c Heatmap displaying differentially expressed genes (≥1.25-fold) with tumor-promoting or tumor-suppressive roles in HCC-1143 cells following RBBP5 knockdown. d qRT-PCR validation of RBBP5-regulated genes identified by RNA-seq. Data are presented as mean ± SD from three technical replicates. Statistical significance was determined using two-tailed Student’s t-tests. ***p < 0.001 compared to the control. shCON, shRNA control; shRBBP5, RBBP5 knockdown. e Western blot showing the expression of WNT5A and key components of the Hippo signaling pathway in HCC-1428 and HCC-1143 cells. CONN, empty vector; RBBP5, RBBP5 overexpression; shCON, shRNA control; shRBBP5, RBBP5 knockdown. f qRT-PCR was performed to assess the mRNA levels of YAP1 target genes in HCC-1428 and HCC-1143 cells. Data are shown as mean ± SD from three technical replicates. Statistical significance was determined using two-tailed Student’s t-tests for HCC-1428 cells and one-way ANOVA followed by an LSD post hoc test for HCC-1143 cells. ***p < 0.001 compared to the controls (CONN or shCON). g Luciferase assay for YAP1/TAZ transcriptional activity. HCC-1428 and HCC-1143 cells were transfected with the YAP1/TAZ-responsive reporter (8xGTIIC-luciferase) for 24 h, followed by a luciferase assay to assess transcriptional activity. Luciferase activity was normalized to *β*-galactosidase activity. Data represents mean ± SD from three independent experiments. Statistical analysis was conducted using two-tailed Student’s t-tests for HCC-1428 cells and one-way ANOVA with an LSD post hoc test for HCC-1143 cells. ***p < 0.001 compared to the controls (CONN or shCON). h Cytoplasmic and nuclear fractionation was performed to evaluate the nuclear translocation of YAP1 in the indicated cell lines. SP1 and *α*-tubulin were used as nuclear and cytoplasmic markers, respectively, to confirm fractionation purity.

WNT5A, a key ligand in the noncanonical Wnt signaling pathway, frequently exhibits dysregulated expression in various cancer types. Previous studies have reported that WNT5A and the noncanonical Wnt pathway can inhibit canonical Wnt signaling through multiple mechanisms.^25^ ^,^ ^26^ Based on this, we initially investigated potential changes in *β*-catenin expression. However, we observed no significant alterations in *β*-catenin levels in the cell lines used in this study (Fig. S4a). Intriguingly, a recent study revealed that WNT5A can activate the Hippo pathway, subsequently suppressing YAP1 activity in prostate cancer cells.^27^ This finding prompted us to explore the potential link between RBBP5, WNT5A, and the Hippo pathway in breast cancer.

We investigated changes in the Hippo pathway in RBBP5-overexpressing HCC-1428 and RBBP5-knockdown HCC-1143 cells. Notably, we observed suppression of the Hippo pathway in RBBP5-overexpressing cells, as indicated by decreased phosphorylation of MST1/2, LATS1, and YAP1, and increased expression of YAP1 and its target genes CCN2 ^28^ and Slug.^29^ Conversely, RBBP5-knockdown HCC-1143 cells showed increased phosphorylation of MST1/2, LATS1, and YAP1, and reduced YAP1 and target gene expression (Figure 3e). Furthermore, in addition to WNT5A and CCN2, which were identified as DEGs in our RNA-seq analysis, changes in mRNA levels of known YAP1 target genes, CCN1,^28^ SOX2 ^30^ and SNAI2,^29^ were observed in response to overexpression or knockdown of RBBP5 (Figure 3f).

To further demonstrate modulation of the Hippo pathway according to RBBP5 expression, we conducted a luciferase assay using the YAP1/TAZ-responsive reporter, 8xGTIIC-luciferase. Results showed that RBBP5 overexpression significantly promoted luciferase activity, indicating increased YAP1/TAZ-responsive promoter activity in HCC-1428 cells, while inhibition of RBBP5 expression resulted in reduced luciferase activity in HCC-1143 cells (Figure 3g). Consistently, the nuclear translocation of YAP1 was enhanced in RBBP5-overexpressing HCC-1428 cells compared to control cells, while reduced in RBBP5-knockdown cells, further confirming that RBBP5 affected YAP1 activation (Figure 3h). Taken together, these results suggest that RBBP5 promotes YAP1 activation by downregulating WNT5A expression.

### Altering YAP1 activity counteracts the changes in cancer stem cell characteristics induced by RBBP5

To further investigate whether Hippo pathway suppression is responsible for increased CSC activity induced by RBBP5 overexpression, we conducted recovery experiments using a YAP1 inhibitor and an MST1/2 inhibitor in RBBP5-overexpressing HCC-1428 and RBBP5-knockdown HCC-1143 cells, respectively.

Treatment with the YAP1 inhibitor, verteporfin, in RBBP5-overexpressing HCC-1428 cells reversed YAP1 and downstream target gene expression levels, while upstream components including MST1/2 and *p*-MST1/2 were not influenced. In contrast, the MST1/2 inhibitor, XMU-MP-1, inhibited the Hippo pathway, as indicated by reduced *p*-MST1/2, *p*-LATS1, and *p*-YAP1, and increased YAP1 and downstream CCN2 and Slug expressions (Figure 4a).

**Figure 4.**
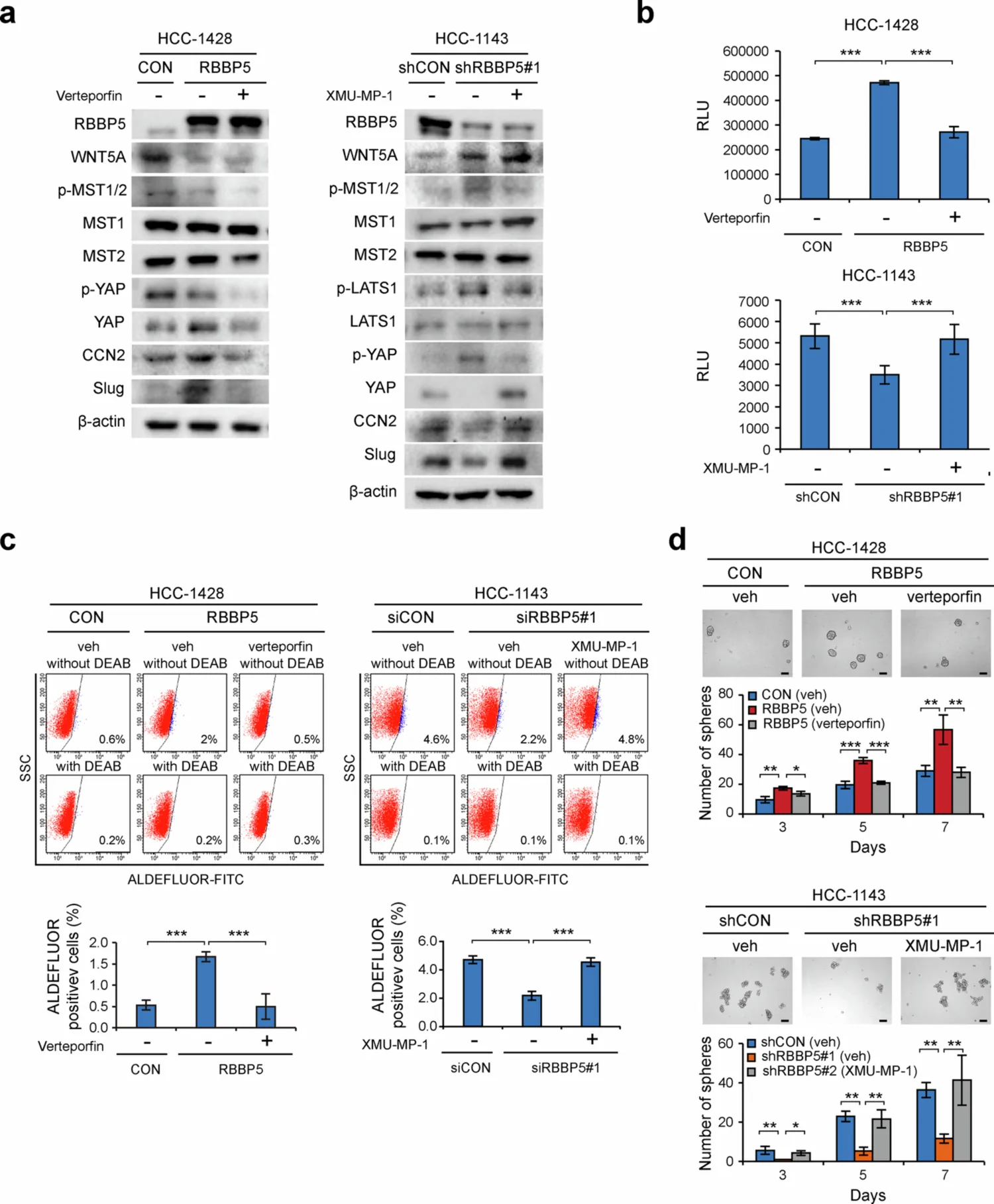
Altering YAP1 activity counteracts the changes in cancer stem cell characteristics induced by RBBP5. a Western blot analysis of lysates from HCC-1428 cells treated with DMSO (vehicle) or 0.5 μM verteporfin for 24 h (left). Western blot analysis of lysates from HCC-1143 cells treated with DMSO (vehicle) or 1 μM XMU-MP-1 for 24 h (right). CONN, empty vector; RBBP5, RBBP5 overexpression; shCON, shRNA control; shRBBP5, RBBP5 knockdown. b Luciferase assay for YAP1/TAZ transcriptional activity. HCC-1428 and HCC-1143 cells were transfected with the YAP1/TAZ-responsive reporter (8xGTIIC-luciferase) for 24 h. HCC-1428 cells were then treated with DMSO (vehicle) or 0.5 μM verteporfin for 24 h, while HCC-1143 cells were treated with DMSO (vehicle) or 1 μM XMU-MP-1 (MST1/2 inhibitor) for 24 h. Data are presented as means ± SD from three independent experiments. *p*-values were determined by one-way ANOVA followed by a post hoc LSD test. ***p < 0.001. c Representative flow cytometry plots and quantification of cell populations with high ALDH activity in the indicated cells. HCC-1428 cells were treated with DMSO (vehicle) or 0.5 μM verteporfin for 24 h, and HCC-1143 cells were treated with DMSO (vehicle) or 1 μM XMU-MP-1 (MST1/2 inhibitor) for 24 h. Following treatment, ALDH activity was assessed using the ALDEFLUOR assay. Data are presented as means ± SD from three independent experiments. *p*-values were determined by one-way ANOVA followed by a post hoc LSD test. ***p < 0.001. CONN, control; RBBP5, RBBP5 overexpression; siCON, siRNA control; siRBBP5, RBBP5 siRNA; with DEAB, DEAB-treated samples; without DEAB, samples not treated with DEAB. d Tumorsphere formation in response to verteporfin or XMU-MP-1 treatment. HCC-1428 cells were treated with DMSO (vehicle) or 0.2 μM verteporfin, and HCC-1143 cells were treated with DMSO (vehicle) or 0.5 μM XMU-MP-1 (MST1/2 inhibitor). Representative images of tumorspheres are shown. scale bars, 100 μm. Quantification data represent the mean ± SD of three biological replicates. Statistical analysis was performed using one-way ANOVA with post hoc LSD tests. *p < 0.05, **p < 0.01, ***p < 0.001.

We further confirmed the effect of inhibitors on YAP1 activity as a transcriptional co-activator through luciferase assay. The enhanced activity of the YAP1/TAZ-responsive promoter observed in RBBP5-overexpressing HCC-1428 cells was reversed by verteporfin treatment. Likewise, RBBP5-knockdown HCC-1143 cells treated with XMU-MP-1 showed recovered promoter activity, exhibiting increased luciferase activity (Figure 4b).

Subsequently, we performed ALDEFLUOR assay and tumorsphere formation assay with these inhibitors. As expected, verteporfin reversed the CSC-like population increased by RBBP5 in HCC-1428 cells, while XMU-MP-1 exerted the opposite effect in RBBP5-knockdown HCC-1143 cells (Figure 4c). Tumorsphere-forming ability assessed by tumorsphere formation assay demonstrated similar results (Figure 4d). In conclusion, our study demonstrated that RBBP5 overexpression confers CSC-like properties through increased YAP1 activity resulting from suppression of the Hippo pathway.

## Discussion

Our results have demonstrated the role of RBBP5 in promoting cancer stem cell (CSC)-like characteristics in breast cancer. Breast cancer patients with high expression levels of RBBP5 are associated with a poorer prognosis. WNT5A expression was downregulated in response to RBBP5 overexpression, resulting in repression of the Hippo pathway and subsequent activation of YAP1. These molecular alterations mediated RBBP5-induced cancer stem cell (CSC)-like properties and were reversed by treatment with a YAP1 inhibitor (Figure 5). These observations suggest that RBBP5 could serve as a potential prognostic biomarker for poor prognosis in breast cancer. Furthermore, modulating the Hippo pathway using pharmacological agents could be a promising therapeutic strategy for breast cancer patients exhibiting RBBP5 overexpression.

**Figure 5.**
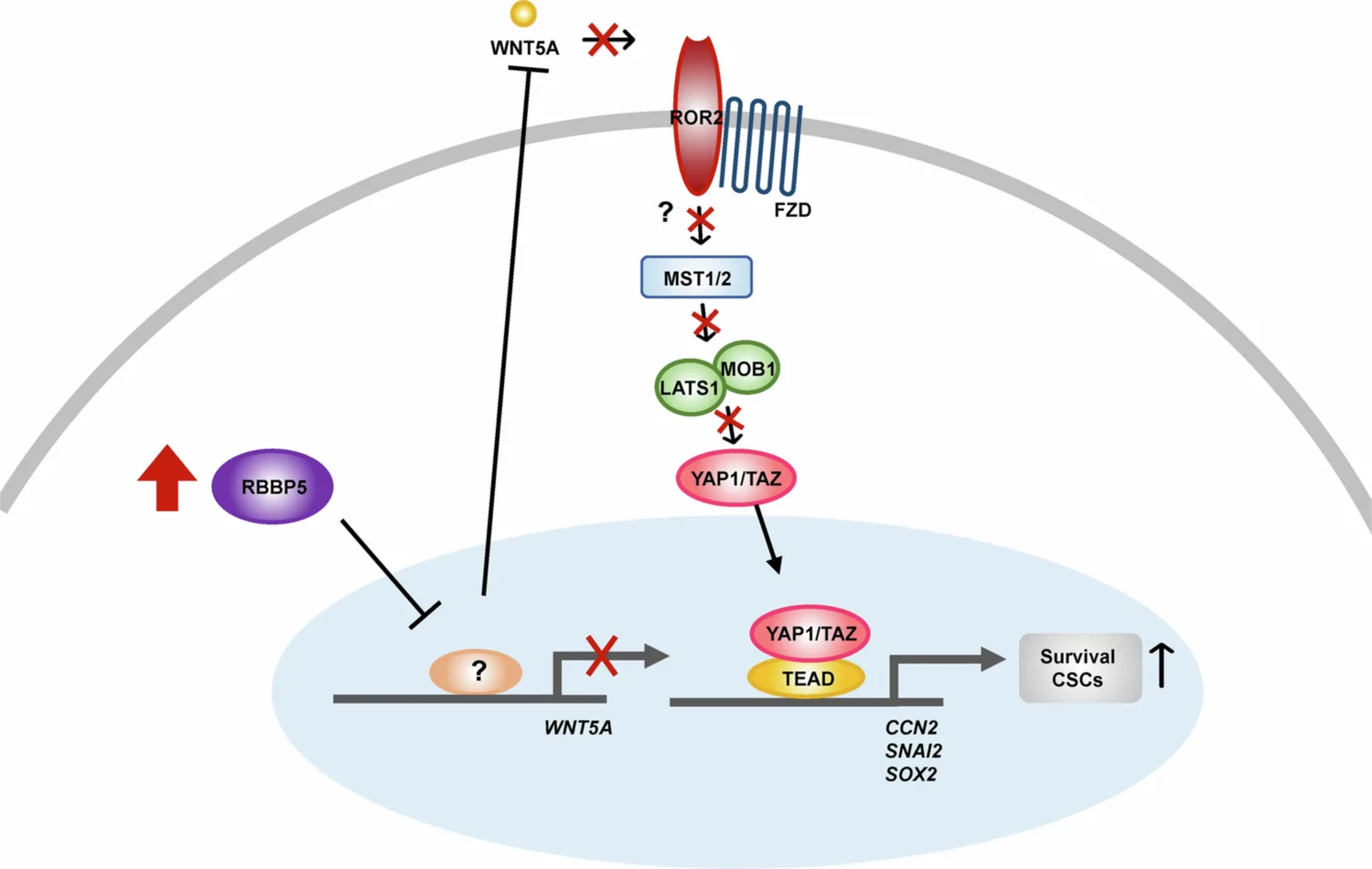
Schematic illustration of RBBP5-mediated regulation of cancer stem cell-like properties in breast cancer. RBBP5 negatively regulates WNT5A expression, which activates the Hippo pathway. Consequently, reduced Hippo pathway activation leads to increased nuclear translocation and activity of YAP1/TAZ transcriptional co-activators. This results in enhanced transcription of YAP1/TAZ target genes, ultimately promoting the acquisition of stem cell-like properties in breast cancer cells.

In this study, we report that RBBP5 is associated with poor prognosis and promotes CSC activity in breast cancer. RBBP5 plays diverse roles in multiple cancers. RBBP5 was upregulated and associated with poor prognosis in hepatocellular carcinoma and glioma.^5^ ^,^ ^6^ Studies have also demonstrated that RBBP5 promotes cell proliferation in various cancer types including nasopharyngeal carcinoma.^5–8^ In breast, prostate, and colorectal cancer, RBBP5 has been implicated in epithelial-mesenchymal transition (EMT).^9–11^ In glioblastoma cancer stem cells, increased RBBP5 expression promoted expression of transcription factors involved in pluripotency.^31^ In addition, RBBP5 knockdown was able to decrease FOXQ1-induced stem-like features in human mammary epithelial cells.^9^ Consistent with these findings, our study shows that RBBP5 drives CSC-like characteristics via Hippo pathway suppression, suggesting RBBP5’s involvement in tumor initiation and poor prognosis. Given that CSCs are major drivers of tumor recurrence and resistance to anti-cancer therapies, including chemotherapy, endocrine therapy, and targeted therapy, our findings suggest that RBBP5 could serve as a prognostic marker associated with poor clinical outcomes and may be useful for stratifying patients who could benefit from therapeutic strategies targeting the Hippo pathway.^32–34^ Therefore, combining standard anti-cancer therapies with Hippo pathway modulation in RBBP5-high breast cancers may represent a promising strategy to suppress CSC-like properties, thereby reducing tumor relapse and therapeutic resistance.

Our data showed that the oncogenic role of RBBP5 is achieved by downregulation of WNT5A expression. Interestingly, RBBP5-mediated repression of WNT5A did not appear to involve a canonical MLL complex-associated mechanism involving enhanced H3K4me3 deposition at gene promoters. One possible explanation for these findings is that the DNA methylation status of the promoter of tumor suppressor gene WNT5A may limit the recruitment of MLL complex, thereby restricting H3K4me3 deposition. Although cancer genomes generally exhibit global DNA hypomethylation, promoter CpG islands of tumor suppressor genes, such as *CDKN2A* (p16), are frequently hypermethylated.^35–37^ Promoter CpG island hypermethylation is generally associated with reduced H3K4me3, reflecting the well-established antagonistic crosstalk between DNA methylation and H3K4me3.^38–40^ The WNT5A promoter has also been reported to be frequently methylated in several cancer types, including colorectal cancer and esophageal squamous cell carcinoma, and such methylation has been associated with transcriptional silencing of WNT5A.^41^ ^,^ ^42^ Thus, promoter methylation may limit the recruitment of RBBP5-containing MLL complexes to the WNT5A promoter, which could explain the relatively modest changes in H3K4me3 enrichment despite alterations in RBBP5 abundance. Collectively, these findings suggest that RBBP5 may regulate WNT5A expression through an H3K4me3-independent mechanism. One possibility is that RBBP5 promotes the expression of an intermediate transcriptional repressor that subsequently suppresses WNT5A transcription. However, upstream transcriptional repressors governing WNT5A expression remain largely unknown in breast cancer. Further investigation to systematically identify these novel intermediate regulators will be an important focus of future studies.

WNT5A plays a complex and often contradictory role in various types of cancer. It functions as both an oncogene and a tumor suppressor, depending on the type and stage of cancer and cellular context.^43^ ^,^ ^44^ As a tumor suppressor, WNT5A negativity was shown to be associated with poor prognosis in hepatocellular carcinoma, and WNT5A overexpression inhibited invasiveness and migration in liver, thyroid, and colon cancers.^45^ ^,^ ^46^ WNT5A also inhibited migration and invasion in breast cancer, partly through downregulation of CD44 expression.^47^ WNT5A repressed tumor growth via Hippo pathway activation in an ROR2-dependent manner in prostate cancer.^48^ In agreement with these findings, we propose that WNT5A exerts its tumor-suppressive effects by activating the Hippo pathway, thereby suppressing YAP1 activity in breast cancer.

Our findings suggest that suppressed Hippo pathway is crucial in RBBP5-induced CSC activity in breast cancer. Numerous studies have investigated Hippo pathway dysregulation and its role in CSCs. Particularly, YAP1 and TAZ, the downstream effectors of Hippo pathway, play crucial roles in regulating CSC properties.^49^ ^,^ ^50^ TAZ expression was shown to be elevated in higher-stage breast cancers and mesenchymal glioma stem cells conferring CSC-like traits in breast cancer and increasing mesenchymal gene expressions in malignant glioma.^15^ ^,^ ^51^ In ovarian cancer, Hippo pathway inactivation through downregulation of MYPT1 led to YAP1-dependent target gene expression and increased stemness.^16^ Additionally, YAP1 promoted stem-like properties in prostate and esophageal cancer cells by regulating downstream target genes such as SOX2, Nanog and SOX9.^17^ ^,^ ^30^ In line with these findings, our study demonstrates that suppression of the Hippo pathway, resulting from RBBP5-induced downregulation of WNT5A, leads to YAP1 activation, thereby promoting CSC-like properties in breast cancer. While we demonstrated that YAP inhibition or activation of the Hippo pathway represents a promising therapeutic strategy, particularly in RBBP5-overexpressing tumor cells, we acknowledge the potential for off-target effects.^52^ ^,^ ^53^ Therefore, the development of more specific targeting approaches—such as tumor-selective delivery systems or combination strategies—may be necessary to enhance efficacy and minimize unintended consequences.

In conclusion, our study has elucidated a novel mechanism by which RBBP5 promotes CSC-like properties in breast cancer. We have shown that downregulation of WNT5A expression by RBBP5 leads to suppression of the Hippo pathway and subsequent increase in YAP1 activity, resulting in acquisition of stem-like characteristics in breast cancer. Future studies will focus on elucidating the precise molecular mechanisms by which RBBP5 downregulates WNT5A expression and the specific signaling events whereby WNT5A induces Hippo pathway activation. Understanding these molecular details will enhance our knowledge of breast cancer stemness and reveal novel therapeutic targets for breast cancer.

## Materials and methods

### Cell culture and reagents

MCF10A (#CRL-10317), BT20 (#HTB-19), MDA-MB-231 (#HTB-26), and HEK293T (#CRL-3216) cells were obtained from the American Type Culture Collection (ATCC, Manassas, VA, USA). JIMT-1 (#ACC 589) cells were purchased from the DSMZ (Braunschweig, Germany). MCF7 (#30022), T47D (#30133), HCC1428 (#9S1428), ZR75-1 (#21500), BT474 (#60062), HCC1954 (#9S1954), SKBR3 (#30030), and HS578T (#30126) cells were purchased from the Korean Cell Line Bank (Seoul, Republic of Korea). Tamoxifen-resistant MCF7 cells (MCF7-TamR) were kindly provided by Prof. Keon Wook Kang (Seoul National University, Seoul, Korea). MCF7, T47D, MDA-MB-231, and HEK293T cell lines were cultured in Dulbecco's Modified Eagle Medium (DMEM; #LM001-05, Welgene, Daegu, Republic of Korea) supplemented with 10% fetal bovine serum (FBS; #S001-01, Welgene) and 1% Penicillin-Streptomycin solution (#LS202-02, Welgene). MCF7-TamR cells were maintained in DMEM under the same conditions, except that the medium was additionally supplemented with 3 μM 4-hydroxytamoxifen (Sigma-Aldrich, St. Louis, MO, USA). HCC1428, ZR75-1, BT474, HCC1954, SKBR3, JIMT-1, BT20, HCC1143, and HS578T cells were cultured in Roswell Park Memorial Institute (RPMI) 1640 medium (Welgene) supplemented with 10% FBS and 1% Penicillin-Streptomycin solution. All cells were maintained at 37 °C in a humidified incubator with 5% CO2 atmosphere. MCF10A cells were maintained in DMEM/F12 medium (#LM002-08, Welgene) supplemented with 5% horse serum (#26050088, Gibco, Grand Island, NY, USA), 0.5 μg/mL hydrocortisone (#H0888, Sigma-Aldrich), 20 ng/mL epidermal growth factor (EGF; #E9644, Sigma-Aldrich), and 10 μg/mL insulin (#91077C, Sigma-Aldrich). All cell lines were tested for mycoplasma contamination using the e-Myco™ Plus Mycoplasma PCR Detection Kit (#25237, iNtRON Biotechnology, Seongnam, Republic of Korea) and were confirmed to be negative. Cell line authentication was performed by short tandem repeat (STR) profiling. Verteporfin (#HY-B0146) and XMU-MP-1 (#HY-100526) were purchased from MedChem Express (Monmouth Junction, NJ, USA).

### Establishment of stable cell lines

To establish RBBP5-overexpressing cells, human RBBP5 cDNA was cloned into the lentiviral pLVX-puro vector obtained from Clontech (Mountain View, CA, USA). For generating RBBP5-knockdown cell lines, RBBP5 short hairpin RNAs (shRNAs) (cat. no: RHS4531-EG5929; Clone IDs: shRNA #1: V3LHS_326589 and shRNA #2: V3LHS_326585) were inserted into the pGIPZ vector (GE Dharmacon, Lafayette, CO, USA). Lentiviral particles containing RBBP5 cDNA and shRNA were produced as previously described ^54^ and used to infect each breast cancer cell line with 6 μg/mL polybrene (#107689, Sigma-Aldrich). To select successfully infected cells, the cells were maintained in media containing 2 μg/mL puromycin (#P7255, Sigma-Aldrich) for 7 days.

### Transfection of siRNAs

Small interfering RNAs (siRNAs) targeting RBBP5 (#1: 5’-GUGUGAAAAGGGCUCAGUAtt-3’, cat. no. 5929-1; #2: 5’-CAGAUUCUCAGGAUCUUGUtt-3’, cat. no. 5929-2) were purchased from Bioneer (Daejeon, Republic of Korea) and transfected into breast cancer cells using RNAi Max (Invitrogen, Carlsbad, CA, USA) for 24 hours.

### ALDEFLUOR assay

Aldehyde dehydrogenase (ALDH) activity was assessed using the ALDEFLUOR kit (#01700, Stem Cell Technologies, Cambridge, MA, USA) according to the manufacturer’s protocol. Briefly, cells were harvested and resuspended in ALDEFLUOR assay buffer at a concentration of 1 × 10^6^ cells/mL. The ALDH substrate, BODIPY-aminoacetaldehyde (BAAA), was added to the cell suspension, and 5 × 10^5^ cells were immediately transferred to a negative control tube containing diethylaminobenzaldehyde (DEAB), a specific ALDH inhibitor. Both the negative control and experimental samples were incubated at 37 °C for 20 minutes in the dark. After incubation, cells were centrifuged, resuspended in ALDEFLUOR assay buffer, and filtered through a 40-μm cell strainer to remove cell aggregates.

Flow cytometry analysis was performed using a FACS Diva (BD Biosciences). Forward scatter (FSC) and side scatter (SSC) parameters were used for gating to exclude doublets and non-viable cells. An SSC vs. ALDEFLUOR (FITC) plot was generated to identify cells with high ALDH activity.

### Tumorsphere formation analysis

MCF7 (5 × 10^3^ cells/well), T47D (5 × 10^3^ cells/well), HCC-1428 (1 × 10^4^ cells/well), tamoxifen-resistant MCF7 (TamR) (3 × 10^3^ cells/well) cells were plated in 6-well ultra-low attachment surface plates (Corning, NY, USA) in DMEM-GlutaMAX medium (#10569-010, Gibco) supplemented with 2% B27 (#17504-044, Gibco), 4 ng/mL heparin (#H3393, Sigma-Aldrich), 20 ng/mL EGF (#E9644, Sigma-Aldrich), and 10 ng/mL basic fibroblast growth factor (bFGF; #100-18B, Thermo Fisher Scientific, Waltham, MA, USA). HCC-1143 (1 × 10^4^ cells/well) cells were seeded under the same conditions as other cell lines, except that the medium for HCC-1143 did not include heparin. DMEM-GlutaMAX medium for ZR75-1 (1 × 10^4^ cells/well) cells was supplemented with 2% B27, 4 ng/mL heparin, 20 ng/mL EGF and 10 ng/mL bFGF. Tumorsphere formation was assessed and quantified using an inverted microscope after 3, 5, and 7 days of culture.

### Orthotopic xenografts

For the limiting dilution assay, female NOD/SCID mice (5 weeks old) were obtained from KOATECH (Pyeongtaek, Republic of Korea). After a one-week acclimation period, mice were subcutaneously implanted with 17β-estradiol (E2) pellets (0.72 mg/pellet, 60-day release; Innovative Research of America, Sarasota, FL, USA). Seven days post-implantation, Control (CONN) or RBBP5-overexpressing (RBBP5) MCF7 cells and control (shCON) or RBBP5-knockdown (shRBBP5) ZR-75-1 cells were serially diluted and resuspended in a 1:1 mixture of phosphate-buffered saline (PBS) and Matrigel (#354234, Corning, NY, USA) to a final volume of 100 μL. The prepared cell suspensions were then orthotopically injected into the mammary fat pads of the mice (*n* = 7 per group).

Tumor growth was monitored twice weekly for 4-7 weeks using calipers. Tumor volume was calculated using the formula: V = 1/2 × (length) × (width)^2^, where length represents the longer dimension. Only masses with volumes of ≥ 30 mm^3^ were counted as tumors. The frequency of tumor-initiating cells (TICs) was determined using the L-Calc software (Stem Cell Technologies, https://www.stemcell.com).

The sample size of *n* = 7 per group was chosen based on earlier research and was deemed adequate for identifying biologically significant variations in tumor growth with appropriate statistical power while minimizing animal use.^55^ ^,^ ^56^ Humane endpoints were defined according to the Hanyang University IACUC guidelines. Specifically, mice were humanely euthanized via CO_2_ inhalation followed by cervical dislocation if they exhibited impaired mobility or feeding, severe distress, > 20% body weight loss, or when tumor burden exceeded 10% of body weight.

### Western blot analysis

Cells were lysed using Radio-Immunoprecipitation Assay (RIPA) buffer (50 mM Tris, pH 7.4, 1 mM EDTA, 150 mM NaCl, 0.25% sodium deoxycholate, 1% NP-40) supplemented with a protease inhibitor (#11873580001, Sigma-Aldrich) and phosphatase inhibitors (#P5726 and #P0044). The samples were heated at 95°C for 5 min and then separated using 8-10% SDS-polyacrylamide gel electrophoresis (SDS-PAGE) and transferred to nitrocellulose membranes. After blocking with 5% non-fat dry milk, the membranes were incubated overnight with primary antibodies at 4°C. The membranes were then subjected to repeated washing and subsequently incubated with secondary antibodies at room temperature for 1 hour. After repeated washing, protein bands were detected using the ECL (Enhanced Chemiluminescence) substrate (#EOE003S, Enzynomics, Daejeon, Korea).

The following antibodies were used for western blot: RBBP5 (#13171, 1:5000 dilution), MST1 (#3682S, 1: 1000 dilution), MST2 (#3952S, 1: 1000 dilution), phosphorylated (*p*)-MST1/2 (#49332S, 1: 1000 dilution), LATS1 (#3477S, 1:3000 dilution), *p*-LATS1 (#8654S, 1:3000 dilution), YAP (#14074S, 1:3000 dilution), *p*-YAP (#13008S, 1:5000 dilution) and Slug (9585s) were purchased from Cell Signaling Technology (Beverly, MA, USA). WNT5A (#sc-365370, 1:500 dilution) and CCN2 (#sc-365970, 1:500 dilution) were obtained from Santa Cruz Biotechnology (Dallas, TX, USA). *β*-actin (#MAB1501R, 1:10000 diluition) was obtained from Millipore (Billerica, MA, USA). *α*-tubulin (#GTX628802, 1:20000 dilution) and SP1 (#GTX110593, 1:20000 dilution) were purchased from GeneTex (Irvine, CA, USA). *β*-catenin (#F5682, 1:3000 dilution) was obtained from BD Bio sciences (Franklin Lakes, NJ, USA).

### Quantitative real-time polymerase chain reaction (qRT-PCR)

Total RNA was extracted from cells using TRIzol reagent (#15596018, Invitrogen, Carlsbad, CA, USA), and cDNA synthesis was performed using the Reverse Transcription System (#A5003, Promega, Madison, WI, USA). qRT-PCR was carried out using a CFX Connect Real-Time PCR Detection System (Bio-Rad, Hercules, CA, USA) with SYBR Green master mix (#4368708, Applied Biosystems, Foster City, CA, USA). The data were normalized to the mRNA levels of GAPDH. The primer sequences used for qRT-PCR are provided in the Supplementary Information (Table S1).

### Chromatin Immunoprecipitation quantitative real-time PCR (ChIP-qPCR) assay

ChIP-qPCR assays were performed as previously described.^18^ Briefly, cells were crosslinked with 1% formaldehyde for 10 min at 37 °C and quenched with 0.125 M glycine. After harvesting and resuspension in Sodium Dodecyl Sulfate (SDS) lysis buffer (Cosmo Bio Co. Ltd., Tokyo, Japan), cells were sonicated using a Bioruptor. The lysate was then precleared with ChIP dilution buffer and salmon sperm DNA coupled with protein A-agarose (#16-157, Millipore) for 2 h at 4 °C. The precleared lysate was incubated with specific antibodies against RBBP5 (#13171, Cell Signaling Technology), H3K4me3 (#Ab8580, Abcam, Cambridge, UK) or control IgG (#GTX35035, GeneTex) overnight at 4 °C. Salmon sperm DNA coupled with protein A-agarose was added to capture the immune complexes. After 2 h of incubation at 4 °C, the complexes were washed and eluted. To reverse crosslinks, 5 M NaCl was added, and the eluates were incubated at 65 °C overnight. Purified DNA fragments were dissolved in ddH2O and subjected to qPCR to analyze the enrichment of ChIP signal (signal/noise ratio). The following specific primers were used: WNT5A promoter (forward: 5′-CAGGCTTAACCCGGTCGCT-3′ and reverse: 5′-CCACCGCAACTCCTGGGCTTA-3′).

### Cytoplasmic and nuclear fractionation

Cytoplasmic and nuclear fractionation was performed using the NE-PER Nuclear and Cytoplasmic Extraction Reagents (#78833, Thermo Fisher Scientific) following the manufacturer’s protocol. The extracts of each cytoplasmic fraction and nuclear fraction were subjected to western blot to assess nuclear translocation of YAP. The cytoplasmic and nuclear extracts were subjected to western blot analysis to assess YAP nuclear translocation. The quality of fractionation was verified using *α*-tubulin as a cytoplasmic marker and SP1 as a nuclear marker.

### Luciferase assay

For the luciferase assay, the YAP/TAZ-responsive reporter 8xGTIIC-luciferase was obtained from Addgene (plasmid #34615).^57^ HCC-1428 (2 × 10^5^ cells/well) and HCC-1143 (8 × 10^4^ cells/well) cells were seeded in 12-well plates and co-transfected with the 8xGTIIC-luciferase reporter and a *β*-galactosidase expression vector. After 24 h, cells were lysed and luciferase activity was measured using a luciferase assay kit (#E1501, Promega, Madison, WI, USA) according to the manufacturer’s instructions. *β*-Galactosidase activity was determined using a *β*-Galactosidase enzyme assay system (#E2000, Promega). Luciferase activity was normalized to *β*-galactosidase activity and expressed as relative light units.

### RNA sequencing (RNA-seq) analysis

RNA-sequencing analysis was performed on control and RBBP5-knockdown HCC1143 cells by LAS Inc. (Gimpo, Republic of Korea). TRIzol reagent was used for total RNA extraction. Skewer ver 0.2.2 was utilized to trim potential sequencing adapters and low-quality bases from the raw reads. Subsequently, the resulting high-quality reads were aligned to the reference genome using STAR ver 2.5 software. Illumina’s TruSeq Stranded mRNA Sample Preparation Kit was employed to prepare strand-specific sequencing libraries. All samples underwent sequencing using the NovaSeq 500 system (Illumina, San Diego, CA, USA).

For quantification of mapped reads and conversion to gene expression values, Cuffquant in Cufflinks ver 2.2.1 was employed with strand-specific library and default options. The hg38 reference genome annotation from UCSC genome (https://genome.ucsc.edu), provided in GTF format, was utilized as the gene model. The expression values were calculated in Fragments Per Kilobase of transcript per Million fragments mapped (FPKM) unit.

To identify differentially expressed genes between the two biological conditions under study, Cuffdiff in the Cufflinks package with the strand-specific library option was used. Genes exhibiting at least a 1.25-fold change in expression between RBBP5-knockdown and control groups, with a *p*-value < 0.05, were designated as differentially expressed genes (DEGs). These DEGs were further categorized using version 2023.2.Hs of Gene Set from Molecular Signature Database (MSigDB v2023.2.Hs; http://software.broadinstitute.org/gsea/msigdb/index.jsp, accessed on 4 Dec 2023) using version 4.2.3 of Gene Set Enrichment Analysis (GSEA).

### Public data

The breast cancer patient datasets from The Molecular Taxonomy of Breast Cancer International Consortium (METABRIC) ^19^ and The Cancer Genome Atlas (TCGA) ^20^ were obtained from cBioPortal (http://www.cbioportal.org/) for comprehensive survival analysis. These publicly available datasets were reanalyzed using Cancer Target Gene Screening (CTGS; http://ctgs.biohackers.net)^58^ to evaluate both overall survival and disease-free survival. CTGS automatically calculated the optimal cut-off points that yielded the most statistically significant (lowest *p*-value) survival curves, stratifying patient groups based on gene expression levels. Relapse-free survival analysis, focusing on RBBP5 expression (205169_at), was performed using the auto-select best cut-off option in KM plotter (http://www.kmplot.com).^21^ The relationship between RBBP5 copy number alterations and expression levels in both METABRIC and TCGA datasets was examined using CTGS. The Cancer Cell Line Encyclopedia (CCLE) dataset was accessed through cBioPortal. Additionally, OncoPrints illustrating genetic alterations of relevant genes in the TCGA and METABRIC cohorts were generated by cBioPortal.

### Statistical analysis

Statistical analyzes were conducted using various methods to assess the significance of experimental results. To compare differences between two groups, the unpaired Student’s t-test was applied. For comparisons involving multiple groups, one-way analysis of variance (ANOVA) was performed, followed by the least significant difference (LSD) post hoc test or Tukey’s post hoc test when equal variance was assumed. Repeated Measures (RM) ANOVA was utilized to analyze data with multiple time points. Survival data were analyzed using the Kaplan-Meier method with log-rank test. Pearson’s correlation coefficient (*r*) was used to measure the linear relationship between variables. All statistical analyzes were conducted in SPSS (SPSS Inc., Chicago, IL). All experiments were performed at least three times independently. All *p-*values were two-sided and *p* < 0.05 was considered statistically significant.

## Supplementary Material

Supplementary Material Supplementary information revision clean.docx

## Data Availability

The data that support the findings of this study are openly available in figshare at https://doi.org/10.6084/m9.figshare.31986483. The RNA-seq data generated in this study have been deposited in the NCBI Sequence Read Archive (SRA) under accession number PRJNA1230819.
