# Supplementary material for: RBBP5 drives cancer stem cell-like characteristics by suppressing the hippo signaling pathway through WNT5A downregulation in breast cancer
Source: Cancer Biol Ther. 2026 Nov 7;27(1):2756150. doi: 10.1080/15384047.2026.2756150 (PMC13757685; doi:10.1080/15384047.2026.2756150)
Supplement: Supplementary file 1 [file KCBT_A_2756150_SM1775.docx]

**Supplementary Information**

**RBBP5 Drives Cancer Stem Cell-like Characteristics by Suppressing the Hippo Signaling Pathway through WNT5A Downregulation in Breast Cancer**

So-Jeong Moon^1,†,^ Young-Hyeon Kye^1,†^, Gu Kong^1, 2,*^

^1^Department of HY-KIST Bio-convergence, College of Medicine, Hanyang University, Seoul, Republic of Korea

^2^Department of Pathology, College of Medicine, Hanyang University, Seoul, Republic of Korea

^†^These authors contributed equally to this work.

***Correspondence:**

Gu Kong, Department of Pathology, College of Medicine, Hanyang University, 17 Haengdang-dong, Seongdong-gu, Seoul, 133-791, Republic of Korea. Phone: 82-2-2290-8251; Fax: 82-2-2295-1091; E-mail: gkong@hanyang.ac.kr

**This file includes:**

Figs. S1-S4

Table S1

**Supplementary Figures**

**Fig. S1** RBBP5 expression across breast cancer cell lines. **a** Box plot displaying RBBP5 mRNA levels in indicated breast cancer subtypes from METABRIC. The vertical bars from the boxes (whiskers) indicate lowest and highest values. The red horizontal lines inside the boxes represent medians. The lower and upper edges of the boxes indicate the 25th and 75th percentiles, respectively. p-values were determined by one-way ANOVA followed by post hoc Tukey tests. n.s.; not significant. **b** Oncoprints from the Cancer Cell Line Encyclopedia (CCLE) dataset illustrating RBBP5 expression levels and copy number alterations (CNA) in various breast cancer cell lines. **c** Western blot analysis of RBBP5 protein expression in the indicated breast cancer cell lines. **d** Western blot validation of RBBP5 overexpression and knockdown efficiency in the indicated cell lines

**Fig. S2** Impact of RBBP5 on tumor growth in vivo. **a** Tumor growth curves from orthotopic xenograft mouse models injected with MCF7 (5×10^3^ cells, left) or ZR-75-1 (5×10⁴ cells, right) cells. Data are shown as mean ± SEM (n = 7 per group). p-values were determined with RM ANOVA. * *p* < 0.05, ** *p* < 0.01. **b and c** Representative images of tumors harvested from mice inoculated with varying cell numbers of (b) MCF7 (CON vs. RBBP5 overexpression) or (c) ZR-75-1 (shCON vs. shRBBP5) cells in the *in vivo* limiting dilution assay. Scale bars, 5 mm

**Fig. S3** Assessment of RBBP5 binding and H3K4me3 levels at the WNT5A promoter. ChIP-qPCR analysis of RBBP5 and H3K4me3 enrichment at the WNT5A promoter in (a) HCC-1428 and (b) HCC-1143 cell lines Data are representative of three independent experiments and are presented as mean ± SD of technical triplicates. Statistical significance was determined using two-tailed Student’s t-tests for HCC-1428 cells and one-way ANOVA followed by Tukey’s post hoc test for HCC-1143 cells. *** p < 0.001 compared to the control (shCON).

**Fig. S4** RBBP5 and its relationship with canonical Wnt signaling. **a** Western blot analysis of β-catenin expression levels in the specified breast cancer cell lines, showing no significant changes in response to RBBP5 expression

**Supplementary Table**

**Table S1.** **List of primer sequences used for qRT- PCR**

| **Gene** | **Direction** | **Sequence (5’→3’)** |
| --- | --- | --- |
| AGER | Forward  Reverse | CACCTTCTCCTGTAGCTTCAGC  AGGAGCTACTGCTCCACCTTCT |
| CXCL3 | Forward  Reverse | CGCCCAAACCGAAGTCATAG  GCTCCCCTTGTTCAGTATCTTTT |
| CXCL8 | Forward  Reverse | GAGAGTGATTGAGAGTGGACCAC  CACAACCCTCTGCACCCAGTTT |
| CCN2 | Forward  Reverse | CTTGCGAAGCTGACCTGGAAGA  CCGTCGGTACATACTCCACAGA |
| RASIP1 | Forward  Reverse | CCTCGTCTTGGACTGGCTAC  TAGTGGCTGAGCAGATGGTG |
| MFNG | Forward  Reverse | ACCTTGTGGTCACCAACTGCTC  GTCATCGTCCACATGGCAGAAC |
| LAMB3 | Forward  Reverse | GCAGCCTCACAACTACTACAG  CCAGGTCTTACCGAAGTCTGA |
| ITGB4 | Forward  Reverse | CTGCGAGCTGCAAAAAGAGG  ATGTCACTCAGAGAGCCGGT |
| GPR87 | Forward  Reverse | GAAAATCCACTTCCCTGCCG  AGGAAACACTTGGGGACGAT |
| OXTR | Forward  Reverse | TCATCGTGTGCTGGACGCCTTT  CGTGAACAGCATGTAGATCCAGG |
| VCAN | Forward  Reverse | TTGGACCTCAGGCGCTTTCTAC  GGATGACCAATTACACTCAAATCAC |
| RERG | Forward  Reverse | CAACCTACCGACACCAAGCAAC  TGTCGTAGACCAGCACAAAGCC |
| WWC2 | Forward  Reverse | CTGATGAGTCTGTGGCTGGAGA  CGGTCTCTACAATGGCTACATCC |
| WNT5A | Forward  Reverse | GGCTGGAAGTGCAATGTCTT  CCGATGTACTGCATGTGGTC |
| KREMEN1 | Forward  Reverse | ACTGTGAGATACCTGCTTGCC  GTTGGACGTTTTACTGGTGCC |
| BTG2 | Forward  Reverse | CTCACCTGCAAGAACCAAGT  TTCCCCAGGTTGAGGTATGT |
| LPAR1 | Forward  Reverse | CTTTGCTGGGTTGGCCTACTT  GCCATGTGCTAACAGTCAGTCT |
| LRIG1 | Forward  Reverse | CCTGGAGTTGGGAGCATTTGA  CCGAATCCTGTTCCGATTGAG |
| ZBTB10 | Forward  Reverse | AGCCACCTGTTGCCTATCCAGA  CACCTCCATCTTGACTAGTGCC |
| SOX2 | Forward  Reverse | GGGAAATGGGAGGGGTGCAAAAGAGG  TTGCGTGAGTGTGCATGGGATTGGTG |
| SNAI2 | Forward  Reverse | GGGGAGAAGCCTTTTTCTTG  TCCTCATGTTTGTGCAGGAG |
| CCN1 | Forward  Reverse | GGAAAAGGCAGCTCACTGAAGC  GGAGATACCAGTTCCACAGGTC |
| GAPDH | Forward  Reverse | GAAGGTGAAGGTCGGAGTC  GAAGATGGTGATGGGATTTC |
